# Using Phytochemicals to Investigate the Activation of Nicotine Detoxification via Upregulation of CYP2A6 in Animal Models Exposed Tobacco Smoke Condensate by Intratracheal Instillation

**DOI:** 10.1155/2018/7635197

**Published:** 2018-12-19

**Authors:** Dayeon Lee, Seung-Beom Seo, Hyun Jeong Lee, Tae-Sik Park, Soon-Mi Shim

**Affiliations:** ^1^Department of Food Science and Biotechnology, Sejong University, 98 Gunja-dong, Kwangjin-gu, Seoul 143-747, Republic of Korea; ^2^Department of Life Science, Gachon University, Bokjung-dong, Sujung-gu, Sungnam-si, Gyeonggi-do 461-701, Republic of Korea

## Abstract

This study examined the efficacy of standardized* Smilax china* L. root extract (SSCR) containing chlorogenic acid on detoxifying nicotine from tobacco smoke condensate (TSC)* in vitro* and* in vivo*. Chlorogenic acid is an identified bioactive component in SSCR by ultraperformance liquid chromatography/photodiode array/electrospray ionization/mass spectroscopy (UPLC/PDA/ESI/MS). HepG2 liver cells and A549 lung cells were carried for measuring ROS and antioxidant enzymes. Sprague-Dawley rats were treated with nicotine by intratracheal instillation (ITI). Cell viabilities by pretreatments of 5, 12.5, and 25, 50 *μ*g SSCR/mL ranged from 41 to 76% in HepG2 and 65 to 95% in A549. Pretreatments of SSCR inhibited TSC-mediated production of reactive oxygen species (ROS) by 8 and 10% in HepG2 and A549 cells, respectively. However, the expression of CAT, SOD1, and AOX1 was downregulated by SSCR in the both cells. The highest conversion of cotinine was observed at 50 *μ*g/mL of SSCR after 120 min of incubation. SSCR upregulated CYP2A6 3-fold in A549 cells regardless of TSC cotreatment. When Sprague-Dawley rats were treated with nicotine by ITI or subjected to SSCR administration for 14 days, the levels of cotinine in urine increased in SSCR treatment only. The cellular level of antioxidant capacity at 10 or 100 mg/kg body weight/day of SSCR treatment was 1.89 and 1.86 times higher than those of nicotine-control. Results suggest that the intake of SSCR can detoxify nicotine by elevating nicotine conversion to cotinine and antioxidant capacity.

## 1. Introduction

Tobacco smoking induces oxidative stress that may cause detrimental health effects like lung damage, hepatocellular damage, and the decrease of antioxidant defenses [1, 2]. Among toxic components, including 4-(methylnitrosoamino)-1-(3-pyridyl)-1-butanone (NNK), nicotine, nitrosamines, polycyclic aromatic hydrocarbons, and aromatic amines [3–6], nicotine is one of the major components in tobacco smoke that can convert to NNK, a group 1 carcinogen by P450 enzyme such as CYP2A13 [7–9]. Nicotine is also metabolized to cotinine by CYP2A6, detoxifying via urine excretion [10, 11]. Enzymes involving nicotine metabolic pathways are mainly found in the liver and lungs [12]. In order to inhibit CYP2A13 enzyme activity, scientists have developed drugs such as methylenedioxy [13]. However, there is a limitation on evaluating the potential drug interaction in case of over abuse. Thus many studies recently examined the detoxifying activities of phytochemicals derived from plants [14–16]. For instance, a previous study reported that polyphenols from* Houttuynia cordata *had the effect of enhancing nicotine conversion to cotinine as well as scavenging ROS [17].

**Figure 1:**
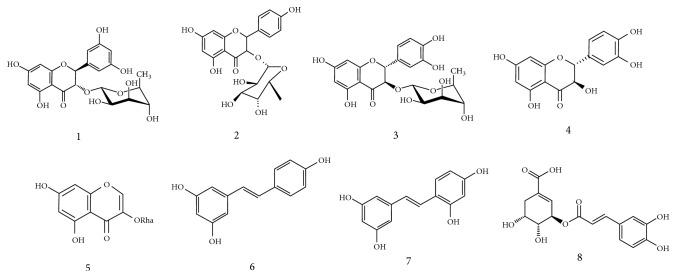
Structures of the eight compounds identified from Smilax china L. (1) smitilbin; (2) engeletin; (3) astilbin; (4) dihydroquercetin; (5) eurryphin; (6) resveratrol; (7) oxyresveratrol; (8) 5-O-caffeoylshikimic acid.

*Smilax china *L. is widely grown in China, Japan, the Philippines, and Korea [10, 18]. Various polyphenols including smitilbin, engeletin, astilbin, dihydroquercetin, eurryphin, resveratrol, oxyresveratrol, and 5-O-caffeoylshikimic acid present in the* Smilax china *L. root are known to contribute useful health effects (Figure 1) [19, 20]. Several studies found these polyphenols provided significant pharmacological activities on inhibiting inflammation, hyperuricemia, and breast tumor [21, 22]. In detail, resveratrol and oxyresveratrol were observed to own strong immune activity on several viruses such as herpes simplex virus (HSV‐1) and human immune‐deficiency virus (HIV) [20].

A previous study reported that the* Smilax china *L. root, including resveratrol and oxyresveratrol as biomarker compounds, showed detoxifying effects on nicotine [10]. Considering susceptible organs are exposed to nicotine when smoking, it is necessary to evaluate the detoxifying effect of the* Smilax china* L. root on lung toxicity. A few studies investigated histopathological toxicity, lung inflammation, and cell proliferation caused by nicotine in the lungs by repeated intratracheal instillation (ITI) administration by using the* in vivo* model [23, 24]. However, there is limited study on preventing nicotine induced liver or lung damage through the consumption of standardized plant extracts by using ITI nicotine exposure in animal models. Thus, we evaluated the detoxifying effect of standardized* Smilax china *L. root extract (SSCR) on nicotine from tobacco smoke condensate (TSC) in hepG2, A549 cells, and animal model exposure and found an enhanced nicotine metabolism and antioxidant defense mechanism.

## 2. Materials and Methods

### 2.1. Standards and Chemical Reagents

Standards of nicotine, cotinine, chlorogenic acid, sodium acetate, potassium cyanide, chloramine T, barbituric acid, sodium metabisulfite, and acetone were purchased from Sigma-Aldrich (St. Louis, MO, USA). All chemicals were of analytical grade. F/12 medium was obtained from the American Type Culture Collection (ATCC, Manassas, VA). Dulbecco's modified eagle's medium (DMEM) and fetal bovine serum (FBS) were purchased from Cellgro (Manassas, VA). Penicillin/streptomycin was obtained from Gibco (Introgen Corporation, Grand Island, NY).

### 2.2. Preparation of* Smilax china *L. Root Extract

*Smilax china *L. roots were acquired from Eumseong-gun, Chungbuk, Republic of Korea, in the spring of 2015. They were washed with purified water and then pulverized to powder for storage in dark conditions. Powder (80 g) of* Smilax china *L. root was extracted in 2 L of 50% ethanol at 55°C with 70 rpm in a shaking water bath for 12 h and then filtered. The sample was concentrated using a rotary evaporator (NE-SERIES, EYELA, Tokyo, Japan) at 60°C with a pressure of 5~7 hpa and a speed of 50 rpm. To avoid exposure to light before further experiments, the SSCR was stored while covered in aluminum foil.

### 2.3. Preparation of Tobacco Smoke Condensate (TSC)

Tobacco smoke condensate (TSC) of 3R4F reference cigarettes was purchased from the Korea Institute of Toxicology (KIT), located in Jeongeup-si, Jeollabuk-do, Republic of Korea. The TSC was made in accordance with the International Organization for Standardization 3308 (ISO 3308). According to previous study [25], total particulate matter (TPM) was filtered from mainstream using a 44 mm Cambridge filter prior to the measurement of TPM and was then mixed in dimethyl sulfoxide (DMSO) at 20 mg TPM mL^−1^. The TSC was filtered using a 0.45 *μ*M PTFE filter and then kept at −80°C. Analytical tests were repeated three times. Nicotine concentration in TSC was 851.01 mg/L. Carcinogenic compounds in TSC were reported to contain 4-(methylnitrosamino)-1-(3-pyridyl)-1-butanone, N-Nitrosoanabasine, N-Nirosoanatabine, and N-Nitrosonornicotine [9].

### 2.4. Identification and Quantification of Bioactive Components in* Smilax china *L. Root Extracts by UPLC-ESI/MS

The concentrate of* Smilax china *L. root extracts was dissolved in 50% ethanol, and 2 *μ*L of this was injected to an ultraperformance liquid chromatography (UPLC) with C_18_ column (Hypersil GOLD, 50 × 2.1 mm, 1.9 *μ*m) at a flow rate of 0.2 mL min^−1^ into a UV detector set at 320 nm wavelength in solvent systems A (0.1% acetic acid in water) and B (Acetonitrile). The gradient of the mobile phase condition was as follows: initial 0–1 min, from A–B (97:3, v/v) to A–B (90:10, v/v); 1–3 min, linear gradient from A–B (90:10, v/v) to A–B (75:25, v/v); 3–5.5 min, linear gradient from A–B (75:25, v/v) to A–B (60:40, v/v); 5.5–6.5 min, linear gradient from A–B (60:40, v/v) to A–B (97:3, v/v); 6.5–8.5 min, maintain gradient to A–B (97:3, v/v). Mass spectra (MS) were accomplished by using a LCQ fleet with electrospray ionization/mass spectrometry (ESI/MS). MS was carried out in a negative ion mode. Other MS conditions were used as follows: capillary temperature (275.00°C), capillary voltage (2 V), aux gas flow (2.00), sheath gas flow (8.00), source voltage (5 kV), and scan range [200~600 (*m/z*)].

### 2.5. A549 Cell Culture

A549 human lung epithelial cells were obtained from the Laboratory of Toxicology at the College of Veterinary Medicine, Seoul National University (Seoul, Republic of Korea). The cells were grown in F/12 supplements with 10% Fetal Bovine Serum (FBS) and 1% penicillin/streptomycin (P/S) at 37°C with 5% CO_2_ and 95% air. They were subcultured when confluence reached 80%. For A549 human lung epithelial cells, the passage used for this study was from 20 to 24.

### 2.6. HepG2 Cell Culture

HepG2 cells were purchased from the American Type Culture Collection (ATCC, Manassas, VA). The cells were sustained in DMEM supplemented with 10% FBS at 37°C in an incubator consisting of 5% CO_2_ and 95% atmosphere. It was subcultured when reaching 80% confluence. For HepG2 cells, the passage used for this study was from 28 to 30.

### 2.7. Measurement of Cell Cytotoxicity by MTT Assay

In accordance with the method reported in a previous study [25, 26], 3-[4,5-dimethylthiazol-2-yl]-2,5-diphenyltetrazolium bromide (MTT) assay was implemented to measure inhibitory effects of SSCR on cytotoxicity induced by TSC. Briefly, HepG2 and A549 cells were randomly plated in a 96-well microplate. After the cells were attached to the 96-well microplate for 24 h, the media was eliminated and then each well was washed using phosphate buffered saline (PBS). The HepG2 and A549 cells were treated by diverse concentrations (5, 12.5, 25, and 50 *μ*g/mL) of SSCR for 6 h, and then cells were treated with IC50 of TSC for 24 h. Following treatment, media was drawn out and each of the cells was added to 100 *μ*L of the MTT solution and then incubated for 4 h at 37°C in complete darkness. After replacing the MTT solution with dimethyl sulfoxide, it was incubated and then the optical density (OD) was determined at 570 nm by using a microplate reader (Varioskan Flash, Thermo Scientific, CA). This operation was repeated three times and the average values were used in the computation as follows:

(1) Percent  of  cell  viability%=Average  of  testO.D.−Average  of  blankO.D.Average  of  controlO.D−Average  of  blankO.D×100

(OD: optical density at 570 nm)

### 2.8. Measurement of Intracellular ROS

In accordance with the method reported in a previous study [25, 27], 2′, 7′-dichlorodihydrofluoresce in diacetate (2′7′-DCFH-DA) assay was conducted to measure inhibitory effects of SSCR on ROS production induced by TSC. Briefly, HepG2 and A549 cells were randomly plated in a 96-well black microplate. After the cells were attached to the 96-well black microplate for 24 h, the media was eliminated, and then each well was washed using phosphate buffered saline. HepG2 and A549 cells were treated by diverse concentrations (5, 12.5, 25, 50 *μ*g/mL) of SSCR for 6 h, and then cells were treated with IC_50_ of TSC for 24 h. After treatment, the media was eliminated and cells were detached by trypsin EDTA and transferred to a 96-well black microplate and consequently treated with 2′7′-DCFH-DA. Lastly, the fluorescence of each cell was measured using a microplate reader (Varioskan Flash, Thermo Scientific, San Jose, CA) at 488 nm for excitation and at 525 nm for emission. This experiment was conducted three times, and the mean values were calculated as follows:

(2) Percent  of  ROS  generation%=Average  of  testAverage  of  control×100

### 2.9. Measurement of Conversion from Nicotine to Cotinine

The amount of cotinine converted from nicotine was measured by the DBA according to the method proposed by Kim et al. (2014) [10]. In brief, when the HepG2 cells reached 100% confluency in a 24-well plate, cell media were removed and media containing 1 mM nicotine were added to the cells. After 24 h of incubation for treatment, SSCR (1, 10, and 50 *μ*g/mL) were added to the cells. They were located in an incubator for 2 h, which is the half-life of nicotine. At various time points (0, 10, 30, 60, 90, and 120 min), cell media were removed and washed by PBS 3 to 4 times. The cells were separated by using trypsin EDTA and then centrifuged for 6 min at 4500 rpm. The supernatant was removed and the cells were washed by PBS. The cells were sonicated for 1 min in 100 *μ*L of PBS and then mixed with 100 *μ*L of deionized water. In order to measure the amount of cotinine, 100 *μ*L of 4 M sodium acetate buffer (pH 4.7), 40 *μ*L of 1.5 M potassium cyanide, 40 *μ*L of 0.4 M chloramin T, and a mixture of 100 *μ*L 78 mM barbituric acid in acetone/water (50:50, v/v) were added to the cells and mixed well for 30 sec. Then it was incubated for 15 min at room temperature before 40 *μ*L of 1 M sodium metabisulfite was added. Absorbance at 490 nm was measured by a microplate reader. The experiment was reiterated three times, and the amount of cotinine was quantified by a standard curve. The results are adjusted with the contents of cellular protein amount.

### 2.10. Measurement of Protein

Protein concentration of the supernatant was measured by the Bradford method (Bradford, 1976). Biochemical measurements were carried out at room temperature using a spectrophotometer (Cecil CE 3041; Cambridge, UK).

### 2.11. RNA Preparation and Quantitative Real-Time PCR

After treatment of TSC or SSCR, total RNA was isolated by a total RNA Extraction Kit (iNtron, Korea, 17-221) according to the manufacturer's procedure. The first strand of cDNA was synthesized from RNA in a PCR Thermal Cycler (Takara, Japan) using the iScript cDNA synthesis Kit (BioRad, Hercules, CA). Real-Time PCR was carried out using a SYBR Green Master Mix (Takara, Japan) using gene-specific primers and 50 ng of cDNA in a StepOnePlus Real-time PCR system (Applied Biosystems, Carlsbad, CA). The primer sequences used in this study were *β*-actin, forward-GGCATCCTCACCCTGAAGTA, reverse-GGGGTGTTG AAGGTCTCAAA; superoxide dismutase (SOD1), forward- GGTGGGCCAAAGGATGAA GAG, reverse- CCACAAGCCAAACGACTTCC; catalase, forward-TGGAGCTGGTAACCC AGTAGG, reverse-CCTTTGCCTTGGAGTATTTGGTA; aldehyde oxidase (AOX1), forward-CTTGGTGGTAACCTGTGCC, reverse-TTCTGCGAAGAGTTTTGGACTT. The PCR conditions were applied as shown previously [27]. The expression of genes was normalized by the relative ratio to *β*-actin.

### 2.12. Animals Study

Thirty male rats of the Sprague-Dawley strain (SD, body weight (BW, 273 ± 7 g), fed with standard laboratory food and water, were used in the study. They were randomly divided into five groups (six rats per group) and placed in separate cages during the study. All animals were kept under the same laboratory conditions of temperature (22 ± 3°C), lighting (12 hr light/12 hour dark cycle, 150–300 Lux), and humidity (50 ± 20%). Our animal study was conducted in an ethically appropriate way, which follows guidelines set by Guide for the Care and Use of Laboratory Animals (an ILAR publication). Nicotine administration was proceeded by using intratracheal instillation (ITI), which has been proposed as the most reliable route for assessing the pulmonary toxicity of particles in rodents [24]. Each group of SD rats was pretreated with SSCR (1, 10, and 100 mg/kg body weight/day) for a week before nicotine (0.2 mg/kg body weight) was treated by intratracheal instillation (ITI) for 14 days. At the same time, three different concentrations of SSCR (1, 10, and 100 mg/kg body weight/day) were administered for 14 days by oral gavage.

The groups were as follows:

- Group 1: control group (received only the same amounts of vehicles, 0.9% NaCl solution)
- Group 2: nicotine group (nicotine 0.2 mg/kg body weight/day, ITI)
- Group 3: low dose group (nicotine 0.2 mg/kg body weight/day, ITI) + SSCR (1 mg/kg body weight/day, oral gavage)
- Group 4: mid-dose group (nicotine 0.2 mg/kg body weight/day, ITI) + SSCR (10 mg/kg body weight/day, oral gavage)
- Group 5: high dose group (nicotine 0.2 mg/kg body weight/day, ITI) + SSCR (100 mg/kg body weight/day, oral gavage)

The urine samples were collected 1 day, 7 days, and 14 days after nicotine ITI treatment. On every day, urine samples were collected a rat in a metabolic cage during 17 h.

Blood from the caudal vein was collected 30 min after nicotine ITI treatment. It was centrifuged at 3000 rpm for 10 min and then the plasma was separated. Both urine and plasma samples were stored at −80°C until analysis.

### 2.13. Quantification of Cotinine from Urine by Using UPLC-UV

The content of urinary cotinine was analyzed according to the method of Petersen et al. (2010) [28]. Briefly, a 1.0 mL urine sample was alkalinized with 50.0 *μ*L sodium hydroxide. The sample was extracted with 5 mL dichloromethane. The sample was dried under nitrogen flow at room temperature and recovered with 100 *μ*L of the mobile phase. Finally, 20 *μ*L of the sample was injected into the HPLC system. The HPLC system was as follows: a HPLC equipped with an isocratic pump, a UV detector, a degasser, and a manual injection system (Model Ultimate 3000, Thermo Science) were used. Chromatographic separations were carried out using a C18 column (150mm x 4.6 mm, 5 *μ*m, Acclaim™ 120), which was protected by a guard column (10 × 4 mm, 5 *μ*m ODS HYPERSIL) and maintained at a temperature of 22 ± 2°C. The mobile phase was a mixture of ultrapure water–methanol–sodium acetate (0.1 M)–ACN (50:15:25:10, v/v/v/v). One milliliter of citric acid (0.034 M) and 5.0 mL of triethylamine were added to each liter of mobile phase. The pH was adjusted to 4.4 with glacial acetic acid. A 0.5 mL/min flow rate was isocratically maintained with the UV set at 261 nm, producing a total run time of 8 min.

### 2.14. Measurement of Radical Scavenging Capacity in Rat Plasma

The oxygen radical absorbance capacity (ORAC) assay was applied to assess the peroxy radical (ROO•) scavenging capacity of rat plasma according to Son and Shim (2015) [29]. For a fluorescent (FL) stock solution, an aliquot amount of the fluorescein sodium salt (44 mg, Sigma-Aldrich) was dissolved with 100 mL of 75 mM phosphate buffer (PB, pH 7.0, Sigma-Aldrich). It was stored in a refrigerator without exposure to light. A 78 mM of the FL working solution was made by dilution with PB and was prepared freshly for each experiment. As an oxidant radical, a specific concentration of 2, 2′-azobis (2-amidinopropane) dihydrochloride (AAPH, Sigma-Aldrich) was prepared by dilution with PB after separation by UPLC. Plasma protein was precipitated with an equal volume of MeOH and centrifuged at 10,000 rpm. A 50 *μ*L of the FL working solution and 50 *μ*L of plasma diluted to 500-fold were added to each 96 well microplate (Corning Inc., Corning, NY, USA). The plate was heated to 37°C for 15 min prior to the addition of the AAPH solution. The fluorescence was measured for an excitation wavelength of 485 nm and an emission wavelength of 535 nm. The relative fluorescence intensity (%) was measured every 5 min by using a microplate reader (Thermo Scientific). It was detected until the value of the fluorescence reading was below 5% of the initial fluorescent light absorbance. The curves of the relative fluorescence intensity of sample could be expressed as the net area under curve (AUC). This was then adjusted to the trolox equivalent (*μ*mol TE) through the Net AUC of samples.

(3) Net  AUC=AUC  of  Fluorescencesample−AUC  of  Fluorescenceblank

### 2.15. Histopathological Studies

Formalin was infused into the lungs via the trachea. The brain, liver, and lung tissues from each animal were preserved in 10% neutral buffered formalin for approximately 48 h before being transferred to 70% ethanol. Preserved tissues were embedded in paraffin wax, sectioned, stained with hematoxylin and eosin, and examined microscopically.

### 2.16. Statistical Analysis

All of the results are expressed as mean ± standard error mean (SEM). The experiment was repeated at least three times. Statistical significance was determined by using a GraphPad Prism 6.0 (GraphPad Software, San Diego, CA, USA) and* p*<0.05 was considered as statistically significant.

## 3. Results

### 3.1. Identification and Quantification of Bioactive Component for Standardization of* Smilax china *L. Root Ethanol Extracts

**Figure 2:**
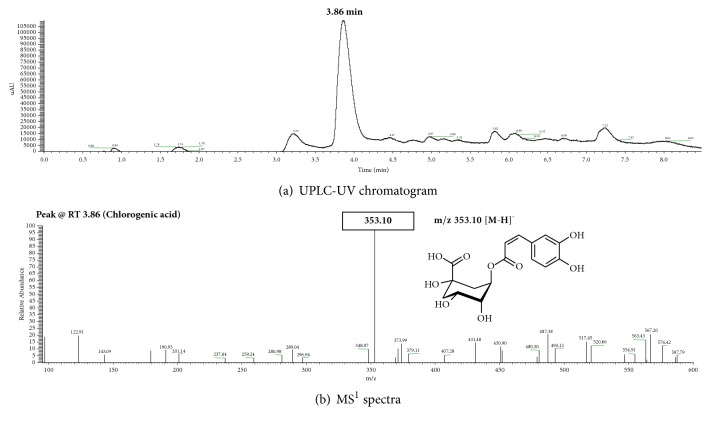
UPLC/PDA/ESI/MS chromatogram of* Smilax china* L. root extracts (SSCR) and identification of major component.

For standardization of* Smilax china *L. root ethanol extracts, the profile of bioactive components in ethanol extracts of* Smilax china *L. root was characterized (Figure 2). As shown in Figure 2(a), one major peak detected at 3.86 min of retention time was identified based on comparison of its retention time with the targeted reference compound and its elution time by UPLC-PDA. Structural identification of a major bioactive component in SSCR was further done according to the molecular ion detected in the negative ion mode by MS (Figure 2(b)). ([M-H]^−^) ion at* m/z *353.10 was obtained from the mass spectra for a major peak, indicating chlorogenic acid. The amount of chlorogenic acid was quantified to be 1.8 g/kg dry weight of SSCR. Previous findings from our group reported that resveratrol and oxyresveratrol were the predominant bioactive components in* Smilax China *L. root, at 267.22 mg/kg fresh weight and oxyresveratrol 68.89 mg/kg fresh weight [10]. The difference in bioactive components in* Smilax china *L. root is attributed to the period and time of collection, origin, soil environment, climate, extraction method, and so on [30]. Thus, standardization of plant extracts on the basis of identification and quantification of bioactive components is necessary [31]. We further examined the targeted biological activity by using standardized* Smilax china *L. root ethanol extracts (SSCR).

### 3.2. Inhibitory Effects of SSCR on Cytotoxicity and ROS Production in HepG2 Cells Induced by TSC

**Figure 3:**
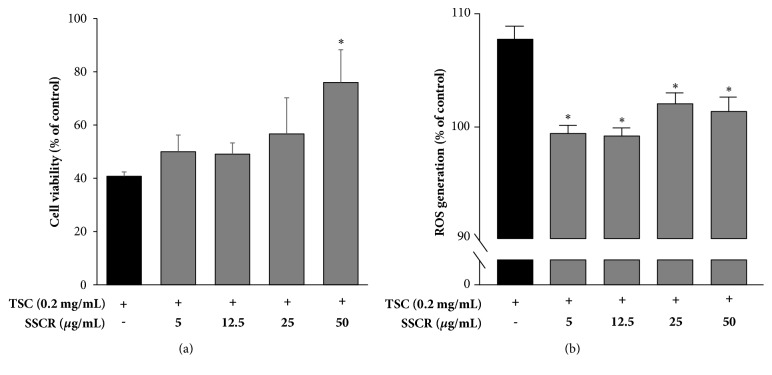
Inhibitory effect of SSCR on cytotoxicity (a) and ROS generation (b) induced by TSC in HepG2 cell. Data are presented as mean ± SEM. *∗p*<0.05 compared with corresponding TSC-only treatment. SSCR, standardized* Smilax china* L. root extracts; TSC, tobacco smoke condensate.

We examined the effect of SSCR pretreatment on cytotoxicity and ROS production in HepG2 cells induced by tobacco smoke condensate (TSC). Cell viability after SSCR treatment was increased at all concentrations in a dose-dependent manner, ranging from 49% to 75% (Figure 3(a)). A remarkable increase in cell viability was found at 50 *μ*g/mL of SSCR pretreatment, implying that a concentration of 50 *μ*g/mL was the optimal concentration for protecting HepG2 cell against TSC exposure. Compared with TSC-only treatment, pretreatment of SSCR on HepG2 cells decreased ROS generation produced by TSC (Figure 3(b)). However, there was no concentration dependency. It suggests that SSCR could protect the liver from TSC induced toxicity by inhibiting cytotoxicity and ROS production. The liver is considered as a major susceptible organ that can be intoxicated by smoking exposure containing various harmful chemicals [2, 32]. In addition, nicotine from smoke extracts is extensively metabolized by CYP2A6 enzymes to several metabolites in the liver, causing liver damage [33]. Previous studies reported that adequate intake of phytochemicals reduced liver damage by scavenging free radicals [10, 21, 34]. For instance, Kim et al. (2014) reported that ethanol extract of* Smilax china* L. root, rich in resveratrol and oxyresveratrol, remarkably decreased nicotine induced ROS production by 11% compared with those of the control. Results from our study showed a similar pattern on enhancing cell viability and removing ROS [10].

### 3.3. Inhibitory Effects of SSCR on Cytotoxicity and ROS Production in A549 Cells Induced by TSC

**Figure 4:**
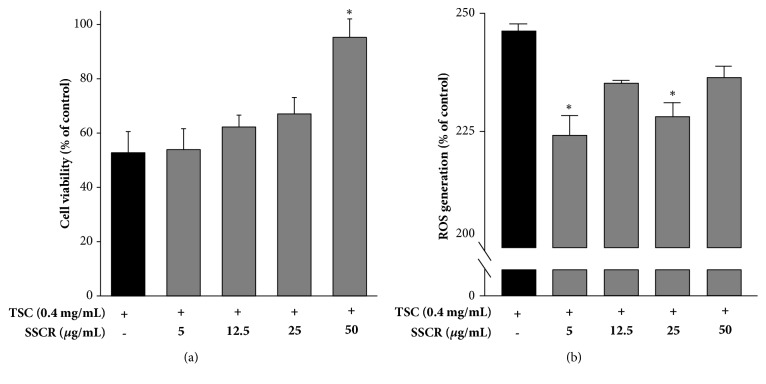
Inhibitory effect of SSCR on cytotoxicity (a) and ROS generation (b) induced by TSC in A549 cell. Data are presented as mean ± SEM. *∗p*<0.05 compared with corresponding TSC-only treatment. SSCR, standardized* Smilax china* L. root extracts; TSC, tobacco smoke condensate.

Since another susceptible target organ that can be directly affected by TSC exposure is the lungs, we further examined the effect of SSCR on A549 cells inhibiting the cytotoxicity and production of ROS by TSC. Pretreatment of SSCR on cytotoxicity and ROS production induced by TSC in A549 cells was examined (Figure 4). The viability of the A549 cells was increased in a dose-dependent manner, ranging from 52.7 to 95.3% from 5 *μ*g/mL to 50 *μ*g/mL of SSCR (Figure 4(a)). When the SSCR concentration was 50 *μ*g/mL, cell viability was significantly enhanced up to 95%. However, there was no significant difference at 5 or 25 *μ*g/mL of SSCR. These results imply that 50 *μ*g/mL of SSCR was the most effective concentration for protecting A549 against TSC exposure as also shown similarly in HepG2 cells. Compared with TSC-only treatment, pretreatment of SSCR reduced ROS generation in A549 cells induced by TSC but the effectiveness was not in a dose-dependent manner (Figure 4(b)). Results from the current study provide a potent SSCR protective effect on lungs against TSC exposure. Cigarette smoke exposure induced production of ROS and disrupts functions of lung epithelium, causing lung inflammation [5, 35]. Plants or their extracts have been shown to provide chemo preventive effects on lungs. For instance,* Ginko biloba* extract downregulates or upregulates diverse signaling pathways and gene transcriptions in the lungs, resulting in treatment of lung inflammation [36]. Considering the results that SSCR could protect cells and reduce ROS production in both HepG2 and A549 cells, we hypothesized that SSCR could modulate gene expression of antioxidant related enzymes in both cells.

### 3.4. Suppression of Antioxidant Enzyme Expression by SSCR in HepG2 and A549 Cells

**Figure 5:**
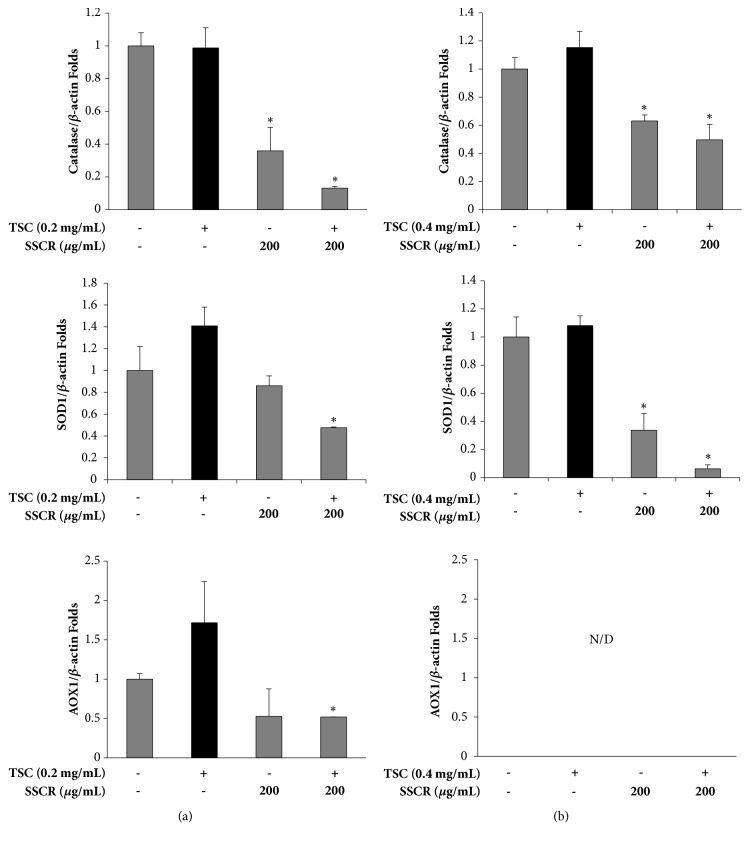
RNA was isolated and real-time PCR was performed to measure the expression of Catalase, SOD1, and AOX1 in (a) HepG2 and (b) A549 cell. The expression was normalized to *β*-actin. The data are presented as mean ± SEM. *∗p*<0.05 compared with corresponding TSC-only treatment. SSCR, standardized* Smilax china* L. root extracts; TSC, tobacco smoke condensate; N/D, Not detected.

We further observed whether ROS scavenging is associated with transcriptional regulation of antioxidant enzymes by measuring gene expression of antioxidant enzymes such as catalase, SOD, and AOX in HepG2 and A549 cells (Figure 5). At 200 *μ*g/mL of SSCR treated with in HepG2, catalase was downregulated regardless of TSC exposure (Figure 5(a)). SOD1 and AOX1 were also downregulated only by coincubation of TSC and SSCR in HepG2 cells (Figure 5(a)). When A549 cells were treated with TSC alone, expression of catalase or SOD1 was not altered (Figure 5(b)). In contrast, SSCR treatment significantly downregulated catalase and SOD in A549 cells. While expression of catalase was not altered in A549 cells, SOD1 was downregulated even further by TSC addition in the presence of SSCR. In A549 cells, expression of AOX1 was not detected (result not shown). Previous studies revealed several mechanisms on scavenging free radicals by natural antioxidants; polyphenols either directly hunt unpaired ROS electrons or indirectly upregulate antioxidant enzymes [37–39]. Taken together, SSCR could reduce oxidative stress and inhibit ROS production by scavenging the ROS with abundant polyphenols in SSCR and not by upregulating the gene expression of antioxidant enzymes in both liver and lung cells.

### 3.5. Effect of SSCR on Conversion from Nicotine to Cotinine in HepG2 Cells

**Figure 6:**
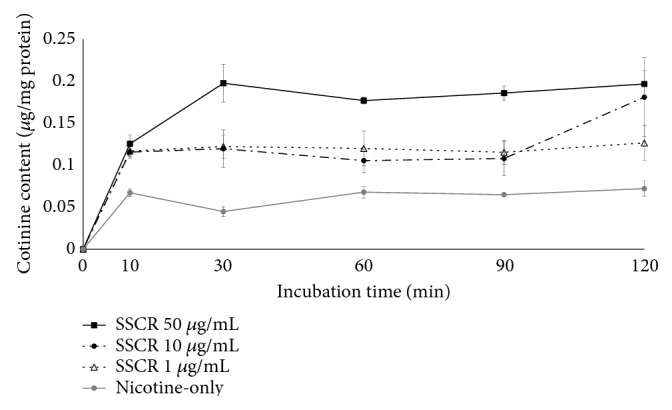
Effect of SSCR on conversion from nicotine to cotinine. The data are presented as mean ± SEM. SSCR, standardized* Smilax china* L. root extracts.

To examine whether SSCR is effective in nicotine conversion with a pathway of cotinine formation, nicotine to cotinine conversion rate was measured according to incubation time in HepG2 cells. The conversion rate of nicotine to cotinine tended to increase at all concentrations of SSCR compared to nicotine-only, showing drastic increases for every 10 min of incubation (Figure 6). It was significantly higher at the concentration of 50 ug/mL concentration of SSCR after 30 min of incubation than other concentrations (p<0.05). The highest cotinine content was 0.19 *μ*g/mg protein at 50 *μ*g/mL of SSCR within half-life of nicotine (120 min). These results imply that SSCR effectively detoxifies nicotine to cotinine in the liver. Nicotine is known to be converted carcinogen, 4-(methylnitrosoamino)-1-(3-pyridyl)-1-butanone (NNK) by p450 enzymes such as CYP2A13 while metabolized to cotinine by CYP2A6 [7–9, 11]. Previous studies revealed that polyphenol in plants, including rutin, quercitrin, and chlorogenic acid enhanced the conversion of nicotine to cotinine, suggesting that it could detoxify nicotine in liver [10, 17, 40, 41]. Similar to our findings, plant extracts containing various polyphenols rather than a single component more effectively promoted conversion of nicotine to cotinine [10].

### 3.6. Induction of CYP2A6 Expression by SSCR in HepG2 and A549 Cells

**Figure 7:**
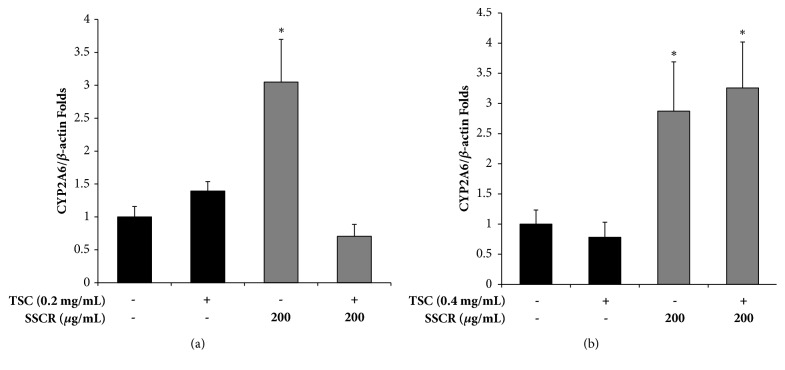
Upregulation of CYP2A6 expression by SSCR in HepG2 (a) and A549 (b). CYP2A6 was normalized to *β*-actin. The data are presented as mean ± SEM. *∗p*<0.05 compared with corresponding TSC-only treatment. SSCR, standardized* Smilax china* L. root extracts; TSC, tobacco smoke condensate.

CYP2A6 is one xenobiotic enzyme that can catalyze the conversion of nicotine to cotinine. We examined whether SSCR regulates the expression of CYP2A6 transcriptionally in HepG2 hepatoma cells (Figure 7(a)) and A549 lung cells (Figure 7(b)). When HepG2 cells were incubated in the presence of TSC for 16 hr, CYP2A6 expression was not altered. However, SSCR upregulated CYP2A6 3-fold (Figure 7(a)). Interestingly, combined treatment of TSC and SSCR nullified this effect (Figure 7(a)). This result suggests that a period of SSCR single treatment as a preventive measure might be necessary to maintain the induced state of CYP2A6. When A549 lung cells were treated in the presence or absence of TSC or in combination with SSCR for 8 hr, TSC-alone did not affect CYP2A6 expression. In contrast, SSCR treatment upregulated CYP2A6 3-fold regardless of TSC cotreatment in A549 cells (Figure 7(b)). Therefore, SSCR has an inducing effect on CYP2A6 expression in liver and lung cells. In addition, A549 lung cells are more sensitive than HepG2 liver cells in maintaining an induced state for CYP2A6 when cotreated with TSC. Another possibility is that SSCR may contain CYP2A6 enzyme-modulatory factors that accelerate the conversion of nicotine to cotinine. However, this possibility is yet to be studied.

Tobacco contains hundreds of known and potential carcinogens that are either activated or detoxified by xenobiotic enzymes, in which cytochrome P450 (CYP) enzymes play an important role in metabolic activation process [42]. In particular, CYP2A6 plays a major role in the metabolism of nicotine to cotinine and the conversion of cotinine to 3′-hydroxycotinine [43]. Similarly, to our results, CYP2A6 activity significantly increased after the consumption of broccoli (500 g/day for 6 days) by 1.4 to 5.5 times. On the other hand, Woodward (2008) suggested that caffeic acid is a plausible inhibitor of nicotine metabolism by inhibiting CYP2A6 [44]. Also, polyphenol interactions with CYP2A6 extend the toxicological effects of nicotine by reducing its rate of removal [45]. However, our results suggest that SSCR induces CYP2A6 transcriptionally and contributes to the activation of nicotine-cotinine conversion.

### 3.7. Histopathologic Examination

The results of the histopathological examination in lungs and livers after a single intratracheal instillation (ITI) dose of nicotine (0.2 mg/kg body weight) are shown in Table 1. Histopathological changes were observed in all groups, but the incidence and severity of the histopathological alterations were not found in the treatment groups, showing no difference from the control group (p>0.05). There were very slight lesions indicated as “P”, indicating that these were considered to be spontaneous or incidental and commonly seen in animal models. There were a few deaths, which are marked by black-filled squares. We confirmed that the ITI dose of nicotine treated in this study would be not enough to induce toxicity in SD rats.

### 3.8. Effect of SSCR on Nicotine Conversion to Cotinine in Animal Models

**Figure 8:**
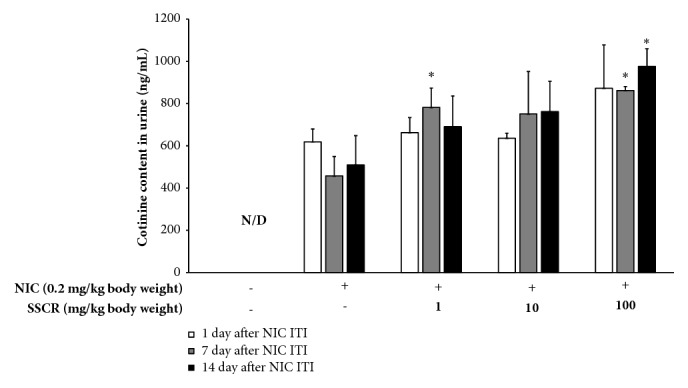
Effect of SSCR on excretion of cotinine in urine from SD rats treated by nicotine ITI and oral administration of SSCR for 14 days. The data are presented as mean ± SEM. *∗p*<0.05 compared with corresponding NIC-only treatment. SSCR, standardized* Smilax china *L. root extracts; NIC, nicotine; ITI, intratracheal instillation; N/D, not detected.

Since nicotine is a major toxic component of cigarette smoke, cotinine in urine and metabolites of nicotine are clinically used as a biomarker for detoxification of smoking [46, 47].  Figure 8 shows changes in cotinine amounts in urine from rats preoral administrated SSCR followed by nicotine ITI for 14 days. The levels of cotinine 30 min after nicotine ITI treatment ranged from 635.9 to 872.7 ng/mL, revealing that there was no significant difference among treatments (*p*>0.05). After 7 days of nicotine ITI with oral administration of SSCR, the detected cotinine amount in urine increased by up to 1.9 times, and it was remarkably high in SSCR preadministration group compared to nicotine only. It was 781.7, 750.9, and 861.9 ng/mL at 1, 10, and 100 mg/kg body weight of SSCR treatment, respectively (*p*<0.05). After the 14 days of nicotine ITI with SSCR oral administration, the measured cotinine amount in urine was 975.7 ng/mL in 100 mg/kg body weight of SSCR treatment, showing a significant difference from nicotine-only treatment (*p*<0.05). These results may imply that pretreatment of SSCR or SSCR + nicotine coadministration effectively metabolized nicotine to cotinine, detoxifying nicotine.

Several studies found comparable data on the preventative effect of plant extracts on various toxicities induced by nicotine in animal models [10, 17, 41, 48]. For instance,* Nigella sativa* oil showed an effect in prevention against nicotine toxicity, enhanced the quality of sperm, and gave better features of testis histology from SD rats chronically treated with nicotine [48]. The current study treated nicotine to animals by ITI rather than oral administration in order to simulate smoking. Thus, to our knowledge, it is the first report on the preventative effect of SSCR on nicotine ITI exposure. Further study still needs to be conducted with long-term exposure by ITI to assess the preventative effect of SSCR on chronic exposure.

### 3.9. Effect of SSCR on Oxygen Radical Absorbance Capacity (ORAC) in Plasma

**Figure 9:**
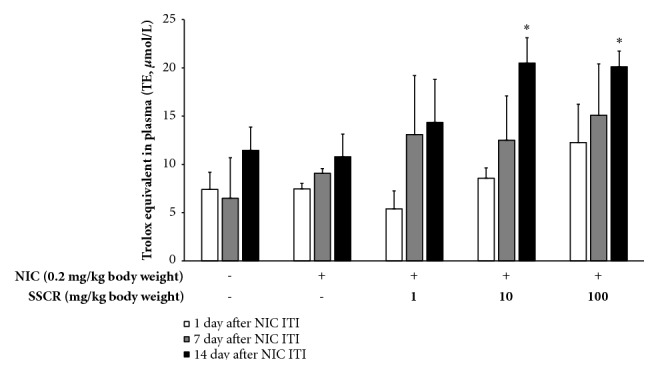
Effect of SSCR on the oxygen radical scavenging capacity in plasma. The data are presented as mean ± SEM. *∗p*<0.05 compared with corresponding NIC-only treatment. SSCR, standardized* Smilax china* L. root extracts; NIC, nicotine; ITI, intratracheal instillation.

Total antioxidant capacity in plasma after consumption of antioxidants has been investigated as an indicator of the antioxidant defense status, showing the ability to elevate circulating antioxidant potentials* in vivo* [49]. Oxygen radical absorbance capacity (ORAC) in plasma from rat administered SSCR with nicotine ITI exposure was measured (Figure 9). Overall, cellular antioxidant capacity was raised in a dose-dependent manner as time passes. After 30 min of nicotine ITI treatment, the antioxidant capacity of plasma ranged from 5.4 to 12.3 *μ*mol/L of trolox equivalent (TE) at 1 to 100 mg/kg body weight of SSCR. However, there was no significant difference compared with nicotine-only treatment (*p*>0.05, Figure 9). On 7th day, all treatment groups of SSCR showed higher antioxidant capacities than nicotine-only treatment groups, but there were no significant differences compared to nicotine-only treatment. However, on the 14th day, the antioxidant capacities were significantly higher at 10 and 100 mg/kg body weight of SSCR treatment, which was 20.5 and 20.1 *μ*mol/L of TE, respectively. Similarly, it was reported that supplementation of plant extracts such as* Hippophae rhamnoides* L. inhibited the malondialdehyde (MDA) in blood from rats exposed to nicotine [50]. Our results imply that various polyphenols including chlorogenic acid in SSCR were absorbed in blood, indicating that the antioxidant capacity in SSCR was shown against nicotine toxicity* in vivo* level.

## 4. Conclusion

In conclusion, results from* in vitro* and* vivo* studies suggest possible pathways for the detoxifying effect of standardized* Smilax china *L. root extracts (SSCR) rich in chlorogenic acid on liver and lung toxicity induced by nicotine from tobacco smoke condensate. First, SSCR directly hunts unpaired reactive oxygen species electrons. Second, SSCR played an important role in transcriptional upregulation of CYP2A6 for conversion of nicotine into cotinine. SSCR could be a useful ingredient for the prevention of diseases related to oxidative stress induced via nicotine or tobacco exposure.

## Figures and Tables

**Table 1:** Histopathologic examination in liver and lung from SD rats. The number indicates the severity of the lesion, ranging from 1 to 5, with 5 being the highest (1=slight, 2=mild). ‘P' indicates a very slight lesion that has been observed but can occur naturally. SSCR, standardized *smilax china* L. root extracts; ITI, intratracheal instillation; SD, Sprague-Dawley strain; asterisk sign mean death.

		**Saline**						**Nicotine ITI (0.2 mg/kg body weight)**																							
H&E = Hematocylin & Eosin staining		**Control **						**Nicotine-only**						**SSCR oral gavage ** **(mg/kg body weight)**																	
														**1**						**10**						**100**					
		**1**	**2**	**3**	**4**	**5**	**6**	**7**	**8**	**9**	**10**	**11**	**12**	**13**	**14**	**15**	**16**	**17**	**18**	**19**	**20**	**21**	**22**	**23**	**24**	**25**	**26**	**27**	**28**	**29**	**30**
**H&E**	**Lung**																														
	Infiltration, mixed cells, alveolus, focal	1	-	-	-	-	-	-	-	-	*∗*	-	-	-	*∗*	-	-	-	-	-	-	-	-	-	-	-	-	-	-	-	-
	Subacute inflammation, alveolus, focal	-	-	-	-	-	-	-	-	-	*∗*	-	-	-	*∗*	-	-	-	2	-	-	-	-	1	-	-	-	-	-	-	-
	Hemorrhage, focal	-	P	-	-	-	-	-	-	-	*∗*	-	-	-	*∗*	-	-	-	-	-	-	-	-	-	-	-	-	-	-	-	-
	Infiltration, eosinophils, perivascular, focal	-	-	1	-	-	-	1	-	-	*∗*	-	-	-	*∗*	-	-	-	-	-	-	-	-	1	-	-	1	-	-	-	-
	Osseous metaplasia	-	-	-	-	-	-	-	-	-	*∗*	P	-	-	*∗*	-	-	-	-	-	-	-	-	-	-	-	-	-	-	-	-
	**Liver**																														
	Infiltration, mononuclear cells, focal		-	-	-	-	1	-	-	-	*∗*	-	1	1	*∗*	-	-	1	-	1	-	1	-	-	-	-	-	-	-	-	1
																														

## Data Availability

The data used to support the findings of this study are included within the article.
